# Hidden Cancer as the Cause of an Ischemic Stroke: A Case Report

**DOI:** 10.7759/cureus.82001

**Published:** 2025-04-10

**Authors:** Yi-Hong Wu, Tung-Chou Li

**Affiliations:** 1 Department of Physical Medicine and Rehabilitation, Cathay General Hospital, Taipei, TWN; 2 School of Medicine, Fu Jen Catholic University, New Taipei City, TWN

**Keywords:** hidden cancer, ischemic stroke, neurorehabilitation, screening, warning signs

## Abstract

Cancer-related stroke is increasingly being recognized as a significant clinical entity. Multiple vascular territory involvement and elevated coagulation markers often serve as key indicators of the underlying malignancy in stroke patients, making their recognition crucial for early cancer detection.

Here, we report a case of a 74-year-old woman who presented with progressive left-sided weakness developing over one week. Brain MRI revealed extensive multifocal acute infarcts involving bilateral cerebral and cerebellar hemispheres. Initial stroke workup showed no conventional risk factors. However, laboratory investigations revealed markedly elevated D-dimer (39.39 μg/mL), decreased fibrinogen (84.3 mg/dL), and elevated fibrinogen degradation product (109.91 μg/mL) levels, consistent with disseminated intravascular coagulopathy. Tumor screening demonstrated significantly elevated tumor markers (CA19-9: 28,960 U/mL, CA125: 394.6 U/mL), and imaging identified a hypervascular ovarian mass. Surgical resection confirmed right ovarian clear cell carcinoma. Postoperative follow-up showed improvement in coagulation parameters, and the patient remained stroke-free for six months under antiplatelet therapy.

This case emphasizes the importance of maintaining clinical suspicion for occult malignancy in patients presenting with cryptogenic stroke, particularly when accompanied by multiple vascular territory involvement and elevated coagulation markers. Early recognition of these warning signs can lead to timely cancer diagnosis and appropriate management strategies, potentially improving patient outcomes.

## Introduction

In Taiwan, cancer is the first and stroke is the fourth leading cause of death [1]. In the United States, about 10% of hospitalized ischemic stroke patients have comorbid cancer, with this association potentially increasing [2]. Furthermore, in the two years following acute ischemic stroke, an additional 3%-5% of patients receive new cancer diagnoses [3]. The most common comorbid cancers in stroke patients are lung, prostate, colorectal, breast, gynecological cancer, lymphoma, and metastatic cancer of unknown primary site [4,5]. Unprovoked venous thromboembolic events, eponymously known as Trousseau's syndrome, raise concern for cancer. Likewise, stroke may be the first clinical sign of an occult tumor [6].

Cancer-related stroke has multiple causes, including tumor procoagulants, cytokines, chemotherapy effects, radiation therapy, and traditional cardiovascular risk factors. The pathogenesis of cancer-related stroke is primarily driven by hypercoagulable states associated with malignancy, including arterial intravascular coagulopathy, nonbacterial thrombotic valve vegetations, and paradoxical embolism, which occurs when deep vein thrombosis (DVT) emboli pass through a patent foramen ovale (PFO) [7,8].

Given that comprehensive cancer investigations are not feasible for all stroke patients, recognizing key indicators of underlying cancer in stroke patients, such as undetermined etiology, multiple vascular territory involvement, and elevated coagulation markers, is crucial for early detection and better patient outcomes [9]. To our knowledge, previous case studies had not comprehensively discussed the red flags for occult cancer in stroke. The current study aims to present a case of cryptogenic stroke with multiple vascular territory involvement in a patient who was subsequently diagnosed with occult malignancy. Also, we report the red flags for a hidden cancer in patients with ischemic stroke, highlighting the importance of maintaining a high clinical suspicion for underlying cancer in stroke patients with specific risk patterns.

## Case presentation

A 74-year-old woman presented with acute progressive left-sided weakness to a tertiary care center. Her medical history included hyperlipidemia managed with rosuvastatin. She reported no prior antithrombotic or herbal use. She lived with her daughter and used a cane for ambulation following vertebroplasty a year ago. Her family history was notable for her father’s stroke in his sixth decade.

**Figure 1:**
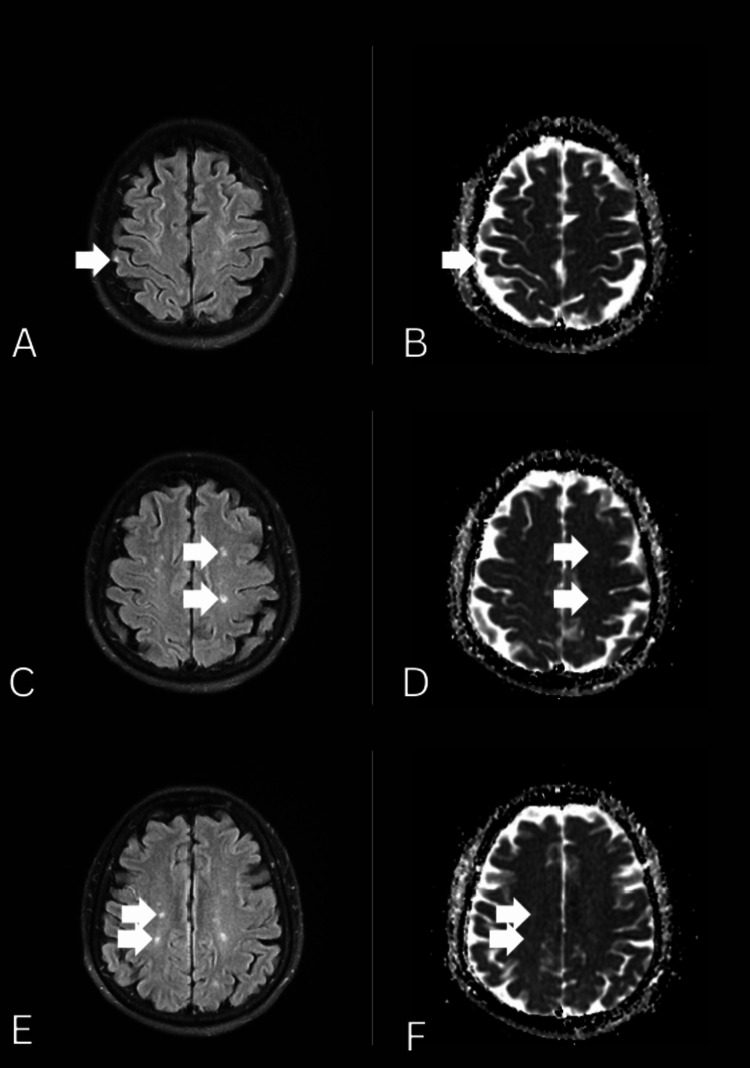
Bilateral cortical and subcortical infarctions Different sections in the axial view of diffusion-weighted imaging (A, C, E) and apparent diffusion coefficient series (B, D, F) showed bilateral cortical and subcortical infarctions (arrows).

One week before admission, the patient experienced transient left body heaviness and unsteady gait. She noted trunk deviation to the left but she did not fall. Three days later, she developed tightness in her right fingers and persistent gait unsteadiness, prompting a visit to another hospital. MRI of the brain revealed extensive multifocal acute infarcts involving the right frontal, bilateral parietal, left temporal, right occipital gray matter, and bilateral cerebellar hemispheres (Figure 1). Clopidogrel was initiated, and she was scheduled for outpatient follow-up.

Four days later, the patient’s left-sided weakness worsened, resulting in a fall. She presented to the emergency department with short-term memory decline. The patient denied sensory loss, muscle twitching, or hallucinations.

On examination, she was hypertensive, with a blood pressure of 159/81 mmHg. She was alert, oriented, and fluent in speech. On assessment, her National Institute of Health Stroke Scale score was 7, attributed to facial palsy, left hemiplegia, and dysarthria. Physical findings were otherwise normal, with no telangiectasia, clubbing, or heart murmurs. Laboratory tests showed mild acute kidney injury (creatinine, 1.38 mg/dL) and mild anemia (hemoglobin, 11.4 g/dL). Prothrombin time and partial thromboplastin time were normal. Non-contrast computed tomography (CT) of the brain revealed a hypodensity in the right temporal lobe. Further investigations to determine the stroke’s etiology were initiated.

**Figure 2:**
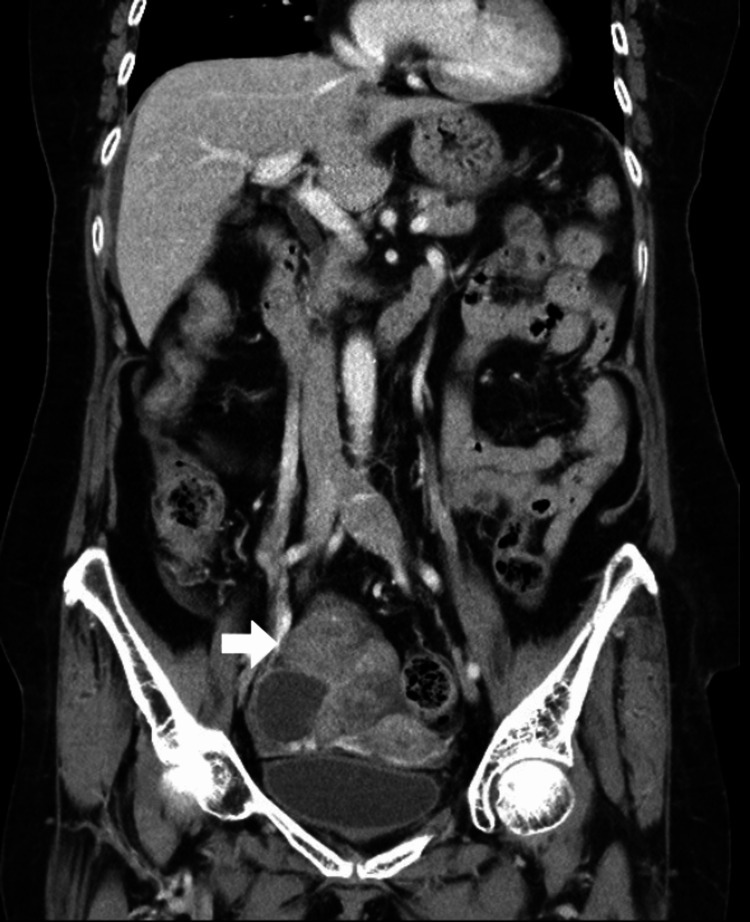
Contrast-enhanced computed tomography of the pelvis A large intraperitoneal lobulated, isodense and heterogeneously enhancing mass (70 x 68 x 58 mm), shown with an arrow, with internal hypoenhancing and cystic components was demonstrated at the right superior aspect of the uterus, with compression on the uterus and superior wall of the urinary bladder.

Blood tests showed a marginally elevated homocysteine level (15.26 μmol/L). Transthoracic echocardiography demonstrated no chamber enlargement or shunting. The 24-hour Holter electrocardiography demonstrated no arrhythmias. Carotid ultrasonography demonstrated no atherosclerosis of intracranial arteries. Hence, suspecting an embolic stroke associated with hypercoagulability, we checked coagulation factors and conducted tumor screening. An elevated D-dimer level was noted (39.39 μg/mL; reference: <0.5). In addition, decreased levels of fibrinogen (84.3 mg/dL; reference: 194-421) and elevated fibrinogen degradation product (109.91 μg/mL; reference: <5) were consistent with disseminated intravascular coagulopathy (DIC). CEA (7.7 ng/mL; reference: <4.9), CA125 (394.6 U/mL; reference: <31.2) and CA19-9 (28,960 U/mL; reference: <36.1) levels were elevated. Contrast-enhanced CT and abdominal ultrasonography revealed a hypervascular ovarian mass measuring 72 × 53 mm, suspiciously malignant (Figure 2).

A surgical staging involving total abdominal hysterectomy, bilateral salpingo-oophorectomy, omental biopsy, and cytological examination demonstrated clear cells, hobnail cells, oxyphilic cells and flat cells with papillary and tubulocystic growth patterns. The diagnoses were right ovarian clear cell carcinoma, pathological International Federation of Gynecology and Obstetrics (FIGO) stage IIA, and cancer-related stroke, with bilateral multifocal infarction.

**Figure 3:**
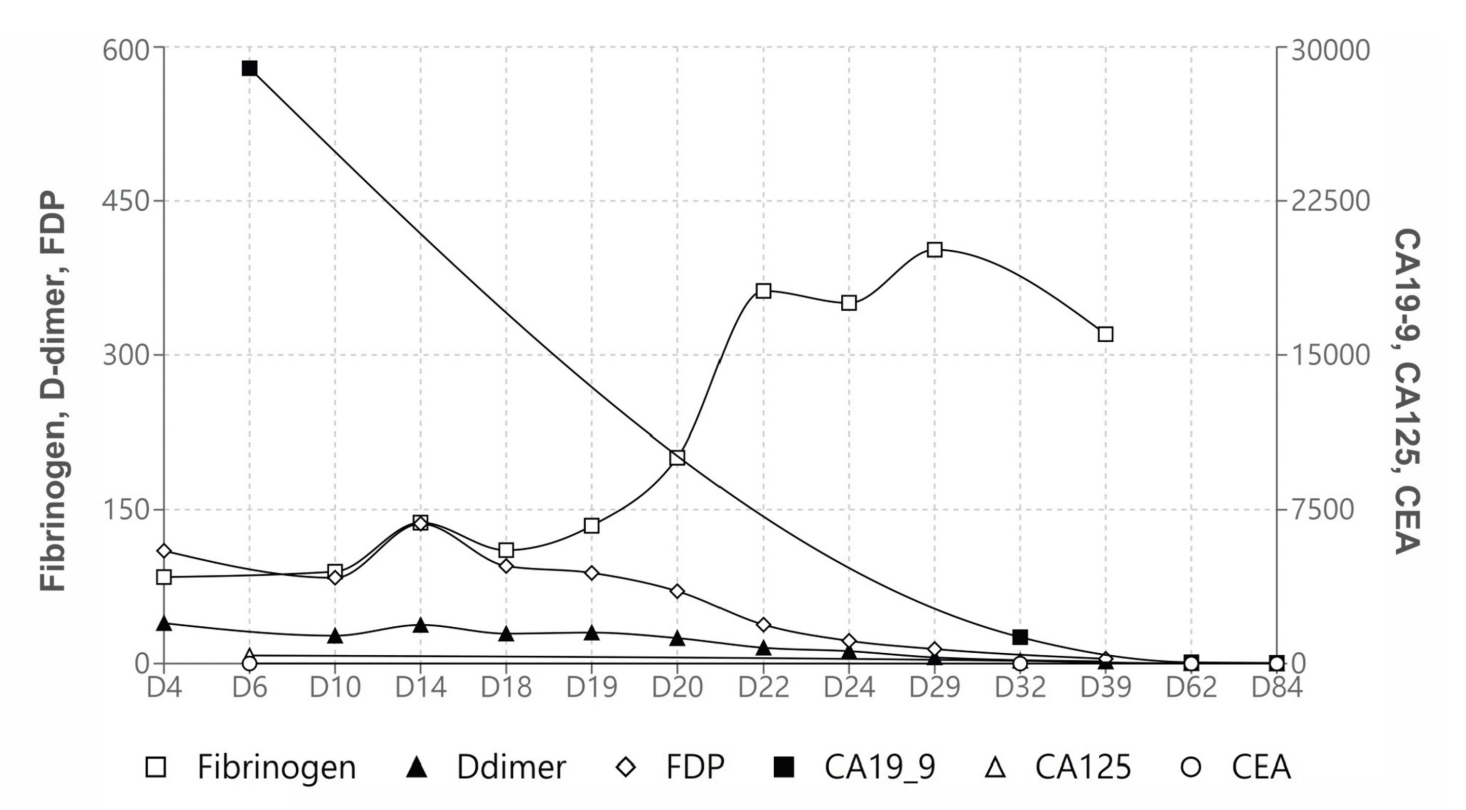
Lab trend of coagulation factors and tumor markers FDP: fibrinogen degradation product

Postoperative laboratory investigations demonstrated a marked improvement in DIC status and coagulation parameters (Table 1, Figure 3). Due to the surgically removed tumor burden, clopidogrel was chosen as secondary prevention. She received physical and occupational therapies, and gained improvements in gait and cognition.

**Table 1:** Lab trend of coagulation and tumor markers after admission FDP: fibrinogen degradation product; INR: international normalized ratio

	Reference range, adults	Day of admission	4 days after admission	6 days after admission	10 days after admission	14 days after admission	18 days after admission	19 days after admission	20 days after admission	22 days after admission	24 days after admission	29 days after admission	32 days after admission	39 days after admission	62 days after admission	84 days after admission
Fibrinogen (mg/dL)	194-421		84.3		89.6	137.3	110.6	134.3	200.5	362.3	350.7	402.4		320.1		
D-dimer (µg/mL)	0-0.5		39.391		27.281	37.599	29.172	30.115	24.844	15.439	12.133	6.034		2.121		
FDP (µg/mL)	0-5		109.91		83.68	136.27	95.08	88.33	70.53	37.95	22.2	14.2		4.67		
INR	0.85-1.15	1.06						1.08	1.03	1.04	0.95	0.89		0.85		
CA19-9 (U/mL)	0-36.1			28960									1290		61	36.4
CA125 (U/mL)	0-31.2			394.6									134.3		9.8	7.1
CEA (ng/mL)	0-4.9			7.7									2.5		2	1.9

## Discussion

Cancer-related stroke differs fundamentally from conventional stroke with mechanisms such as large-vessel disease, small-vessel disease, and cardioembolism. These patients present with a nonlacunar ischemic stroke and no convincing etiology on initial stroke survey, and fall into the category of embolic stroke of undetermined source (ESUS). Notably, the prevalence of cancer-related stroke was shown to reach 3.0% in a Japanese ischemic stroke cohort and 20% in ESUS [10,11]. The distinct etiology and necessitates unique diagnostic and therapeutic approaches.

Cancer-related stroke is predominantly driven by hypercoagulable states inherent to malignancy, with arterial coagulopathy being the most common mechanism [12]. It is therefore understandable that stroke patients with cancer mostly presented with heightened coagulation activity, principally raised D-dimer, fibrinogen monomer, and fibrinogen, along with inflammatory markers such as elevated C-reactive protein (CRP) levels. Hypoalbuminemia and anemia have been linked to cancer following a stroke [4,13]. A probability scoring system for ischemic stroke patients under 75 assigns up to three points based on elevated D-dimer (≥3 mg/L), reduced hemoglobin (≤12.0 g/dL), and a history of smoking (one point each) can guide clinical practice. With a 5% cancer prevalence, the calculated probability of active cancer is 13% for patients scoring two points and 53% for those scoring three, suggesting cancer screening for patients scoring two or more points [13]. Our patient had a D-dimer level of 39.4 ug/mL and hemoglobin at 11.4 g/dL, scoring two points and representing an elevated risk of active cancer.

Studies indicate that elevated D-dimer levels may be linked to cancer-related thrombosis and can be used as a screening tool for malignancy in stroke patients [4,13-15]. Patients with cancer-related stroke have a distinctively elevated level of D-dimer (>20× higher than those without cancer) and multiple lesions in multiple vascular territories [16]. Using a D-dimer cutoff value of >0.55 mg/L with multiple territory infarctions, the specificity and positive predictive value (PPV) for cancer-related stroke were 99.7% and 92.9%, respectively. When the cutoff was set at ≥5.5 mg/L, the test maintained high specificity and PPV, regardless of brain MRI findings, which fits our patient’s presentation [15].

Cancer-related strokes are associated with a poor prognosis. Ischemic stroke recurrence occurs in 21.6% of patients within six months, with elevated D-dimer levels serving as a biomarker for both recurrence and mortality [17]. Notably, even after cancer remission, the risk of recurrent stroke or cardiovascular death remains high, highlighting the need for vigilant long-term management [18]. However, evidence-based protocols specifically for cancer-related strokes are lacking. In summary, direct oral anticoagulants (DOACs) and antiplatelet agents remain the primary treatment options, with no significant difference in mortality or recurrent stroke rates [17,19].

Stroke in cancer patients greatly affects functional status and treatment options. Intensive rehabilitation can restore anti-cancer therapeutic possibilities, but clinicians must evaluate stroke etiology, cancer control status, and patient condition. Rehabilitation wards' limitations in providing concurrent cancer treatment pose challenges. For patients needing extended recovery or with uncontrolled cancer, pursuing intensive rehabilitation requires thorough interdepartmental communication and careful risk-benefit consideration.

## Conclusions

Cancer-related strokes present diagnostic challenges, with unique pathophysiology, poor outcomes, and ongoing risks post-remission. The patient had no new strokes six months after surgery and antiplatelet therapy. However, personalized management based on stroke mechanisms is crucial for optimizing outcomes. This case highlights the importance of considering hidden cancer in stroke patients with multiple vascular territory involvement and elevated coagulation markers.
